# Occurrence and identification of * Colletotrichum* species in mangrove-associated anthracnose

**DOI:** 10.7717/peerj.21307

**Published:** 2026-06-03

**Authors:** Ziyi Ye, Dongmei Cheng, Hua Yang, Changsheng Qin, Jinyan Feng, Longyan Tian, Jinzhu Xu

**Affiliations:** 1 College of Resources and Environment, Zhongkai University of Agriculture and Engineering, Guangzhou, China; 2 Guangdong Provincial Key Laboratory of Silviculture, Protection and Utilization, Guangdong Academy of Forestry, Guangzhou, China

**Keywords:** Mangrove forests, Anthracnose, Morphology, Phylogenetic analysis

## Abstract

Mangrove forests represent vital components of tropical and subtropical coastal wetland, yet recurrent anthracnose epidemics induce foliar necrosis and growth suppression in these ecosystems. To identify causative pathogens and establish a foundation for disease control, this study investigated anthracnose—affected mangrove species (*Bruguiera gymnorrhiza*, * Excoecaria agallocha*, * Rhizophora stylosa*, and * Sonneratia apetala*) in Guangdong Province, China. Through morphological and multilocus phylogenetic analyses (actin (ACT), chitin synthase (CHS-1), glyceraldehyde-3-phosphate d ehydrogenase (GAPDH), internal transcribed spacer (ITS), and β-tubulin (TUB2)), eight * Colletotrichum* isolates were identified: two *C*. *karsti* from the * C*. *boninense* species complex, and six from the * C*. *gloeosporioide*s complex (*C*. *endophyticum* (two isolates), *C*. * pandanicola* (two), *C*. * proteae* (one), * C*. * tropicale* (one)). Pathogenicity assays confirmed all isolates were pathogenic, with *C. endophyticum*, * C. karsti*, and *C. tropicale* exhibiting the highest virulence. Notably, strains * C. endophyticum* Guangdong Microbial Culture Collection (GDMCC) 3.1264, * C. endophyticum* GDMCC 3.1271, * C. karsti* GDMCC 3.1266, and * C. tropicale* GDMCC 3.1265 caused the most severe lesions on * Rhizophora stylosa.* These findings elucidate the etiology of mangrove anthracnose and provide a foundation for targeted biocontrol strategies to conserve coastal ecosystems.

## Introduction

As keystone vegetation formations in tropical and subtropical coastal ecosystems, mangrove communities perform vital ecological functions, including storm surge buffering, carbon sequestration, and shoreline stabilization (Howard et al., 2017). However, synergistic effects of anthropogenic disturbances and climate change have markedly intensified the ecological disruption in these systems (Goldberg et al., 2020). In Guangdong Province alone, 34 distinct mangrove diseases have been documented, including sooty mold and anthracnose (Yang et al., 2025). These pathogens induce chlorosis and defoliation, which severely affect plant growth. Wier & Tattar (2000) reported the widespread dieback of *Rhizophora stylosa* infected by *Cytospora* sp. Zhou & Huang, 2001 identified foliar pathogens from the genera *Alternaria*, *Pestalotiopsis*, and *Phyllosticta* as the primary causal agents in three coastal mangrove reserves of Guangxi Province.

As hemibiotrophic phytopathogens, species of the fungal genus *Colletotrichum* are now recognized as the predominant causative agents of anthracnose in plants (Cannon, Damm & Johnston, 2012). *Colletotrichum* species infect a broad range of plant hosts and exhibit extensive global distribution (Liu, Gao & Li, 2020). To date, nearly 200 plant species have been reported to be susceptible to *Colletotrichum* infections (Marin-Felix et al., 2017). Consequently, this genus is now classified among the most important groups of plant pathogenic fungi worldwide (Liu et al., 2012). Infections typically result in various pre-harvest symptoms, including damping-off, leaf blight, and necrotic spotting. Because of its characteristic latent infection in the field, isolates of *Colletotrichum* can lead to significant post-harvest fruit decay, thereby reducing the yield and market value of economically important crops (Bailey & Jeger, 1992; Anderson et al., 2013).

Anthracnose infection is of particular concern because it affects a large number of different host species and has the potential to cause high levels of disease. *Colletotrichum gloeosporioides* and *C. fructicola* are known to cause anthracnose in *Bruguiera gymnorrhiza* (Huang & Zhou, 1997a; Yuan et al., 2025), while *C. gloeosporioides* also infects *Excoecaria agallocha* (Huang et al., 1997b), and *C. fructicola* is pathogenic to *Sonneratia apetala* (Xiao et al., 2024). These pathogens induce symptoms, such as leaf spots, twig blight, and tissue necrosis, significantly hindering host development and ecosystem function.

This study elucidated the etiology of mangrove anthracnose in Guangdong, China, revealing a diversity of *Colletotrichum* pathogens from the *C. gloeosporioides* and *C. boninense* species complexes. Pathogenicity assays demonstrated significant variation in virulence, with *C. endophyticum*, *C. karsti*, and *C. tropicale* exhibiting high aggressiveness. A critical finding was the strain-specific susceptibility, particularly in *Bruguiera gymnorrhiza*, which was only susceptible to two specific strains. These results provide a crucial foundation for developing targeted biocontrol strategies to mitigate anthracnose and conserve coastal ecosystems.

## Materials and Methods

### Isolation and purification of pathogens

Disease samples exhibiting typical anthracnose symptoms were collected from various mangrove hosts, including *Bruguiera gymnorrhiza*, *Excoecaria agallocha*, *Rhizophora stylosa* and *Sonneratia apetala* at Guangdong Neilingding Futian National Nature Reserve and Zhanjiang Mangrove National Wetland Park in Guangdong Province. To isolate the pathogen, disease samples were sterilized with 75% alcohol for 1 min, and then washed three times with sterilized distilled water. Samples at the junction of healthy and diseased areas were chopped into pieces (about 0.5  × 0.5 cm^2^), and then the pieces were plated on potato dextrose agar (PDA) medium and incubated at 25 °C. After 5 days of incubation, agar blocks were cut from the growing edge of colonies and inoculated onto fresh PDA, and this process was repeated several times to obtain putative pure pathogens.

### Morphological observation

To examine the morphological characteristics of conidia, mycelia plugs were grown onto Potato dextrose agar (PDA) and Oatmeal agar (OA) plates incubated at 25 °C. After 14 days, the sporulation and conidial characteristics were recorded using lactophenol-picric acid solution using an ECLIPSE Ni-U microscope (Nikon) (Su, Qi & Cai, 2012). Fifty conidia were randomly selected for the measurement.

### DNA extraction and PCR amplification

Genomic DNA extraction was performed utilizing the BayBiopure™ Plant Genomic DNA Extraction Kit (Guangzhou BayBio Bio-tech Co., Ltd.) based on magnetic bead separation technology, following the manufacturer’s instructions. Five microliters (5 µL) of DNA solution were used for electrophoresis detection, while residual DNA aliquots were preserved at −20 °C for PCR. Five loci of *Colletotrichum* isolates, including ACT, CHS-1, GAPDH, ITS, and TUB2 were amplified with the primer pairs ACT-512F/ACT-783R (Damm et al., 2012), CHS-79F/ CHS-354R (Carbone & Kohn, 1999), GDF1/GDR1 (Guerber et al., 2003), ITS1/ITS4 (White et al., 1990), and T1/BT-2b (O’Donnell & Cigelnik, 1997; Glass & Donaldson, 1995), respectively. All primers were synthesized by Bioengineering (Shanghai) Co., Ltd. Primer information is shown in Table 1. The total volume of the PCR reaction system was 25 µL, including 12.5 µL of Green Taq Mix, 1 µL of template DNA, 1 µL of forward and reverse primers, and 9.5 µL of ddH_2_O. The reaction procedure is shown in Table 2. The PCR products were detected by 1% agarose gel electrophoresis, and the obtained PCR products were sent to Bioengineering (Shanghai) Co., Ltd. for sequencing.

### Data processing

The construction of the phylogenetic tree was based on the literature related to the identification of *Colletotrichum* (Liu et al., 2022). Sequences of *Colletotrichum* spp. were downloaded from the National Center for Biotechnology Information (NCBI) GenBank database through BLASTn. The details of the sequence information are shown in Table S1. Phylogenetic reconstructions were performed on concatenated sequence datasets using both Maximum Likelihood (ML) and Bayesian Inference (BI) approaches. Date were collected as previously described in Wang et al. (2025a). Specifically, the ML analysis, we employed the GTR + GAMMA substitution model and conducted 1,000 bootstrap replicates using RAxML-HPC v.8, implemented through the CIPRES Science Gateway portal (https://www.phylo.org/; Miller, Pfeiffer & Schwartz, 2010). The Bayesian analysis was performed using partition-specific evolutionary models selected with MrModelTest v.2.3, based on the Akaike Information Criterion (AIC). Markov Chain Monte Carlo (MCMC) simulations were run in MrBayes v.3.1.2 (Ronquist & Huelsenbeck, 2003) with two independent runs of 10 million generations each, starting from random trees. We confirmed run convergence by monitoring the average standard deviation of split frequencies (<0.01) and sampled trees every 1,000 generations. After discarding the first 25% of trees as burn-in, posterior probabilities (PP) were calculated from the remaining trees. Nodal support was assessed using bootstrap support (BS) values (ML) from 1,000 replicates and Bayesian posterior probabilities (PP). The resulting phylogenetic trees were visualised using FigTree v.1.4.4.

**Table 1:** Primers used in this study.

**Gene**	**Primer**	**Sequence (5′–3′)**	**Reference**
ACT	ACT-512F	ATGTGCAAGGCCGGTTTCGC	Damm et al. (2012)
	ACT-783R	TACGAGTCCTTCTGGCCCAT	Damm et al. (2012)
CHS-1	CHS-79F	TGGGGCAAGGATGCTTGGAAGAAG	Carbone & Kohn (1999)
	CHS-354R	TGGAAGAACCATCTGTGAGAGTTG	Carbone & Kohn (1999)
GAPDH	GDF1	GCCGTCAACGACCCCTTCATT	Guerber et al. (2003)
	GDR1	GGGTGGAGTCGTACTTGAGCATGT	Guerber et al. (2003)
ITS	ITS1	TCCGTAGGTGAACCTGCGG	White et al. (1990)
	ITS4	TCCTCCGCTTATTGATATGC	White et al. (1990)
TUB2	T1	GGTAACCAAATCGGTCCTGCTTTC	O’Donnell & Cigelnik (1997)
	Bt2b	ACCCTCAGTGTAGTGACCCTTGGC	Glass & Donaldson (1995)

**Table 2:** The thermal cycling condition for each locus.

**Gene**	**Initial step (T,t)**	**Denaturation (T,t)**	**Annealing (T,t)**	**Elongation (T,t)**	**Cycles**	**Final step (T,t)**
ACT	95 °C, 3 min	95 °C, 30s	58 °C, 30s	72 °C, 45s	34 ×	72 °C, 10 min
CHS-1	95 °C, 3 min	95 °C, 30s	66 °C, 30s	72 °C, 45s	34 ×	72 °C, 10 min
GAPDH	95 °C, 4 min	95 °C, 30s	60 °C, 30s	72 °C, 45s	35 ×	72 °C, 10 min
ITS	95 °C, 3 min	95 °C, 30s	55 °C, 30s	72 °C, 45s	34 ×	72 °C, 10 min
TUB2	95 °C, 3 min	95 °C, 30s	56 °C, 30s	72 °C, 45s	34 ×	72 °C, 10 min

### Pathogenicity test

The pathogenicity test was carried out by *in vitro* inoculation method (Hlaiem et al., 2018). Data were collected as previously described in Li et al. (2023). Specifically, healthy spotless leaves of the year (*Aegiceras corniculatum*, *Bruguiera gymnorrhiza*, *Excoecaria agallocha*, *Rhizophora stylosa* and *Sonneratia apetala*) were rinsed with running water, surface-sterilized with 75% ethanol for 60 s and rinsed three times with sterile distilled water. All inoculated leaves were punctured with a sterile needle on the surface, and plugs (⌀ = 6 mm) of each strain that had been cultured for 7 days were inoculated on the leaf with the mycelial surface down. Leaves inoculated with sterile PDA agar were used as control. Three independent experiments were performed with three biological replicates each. The inoculated leaves were then placed into sterile plastic culture dishes (⌀ = 15 mm) that contained wet paper and sealed with parafilm to remain moist, and placed in a controlled-environment growth chamber under humidity-controlled conditions (25 °C, 12 h light; 23 °C, 12 h dark). The disease development of the leaves was continuously observed, and the pathogen on the diseased leaves was re-isolated, identified and compared with the original inoculated strain to verify whether Koch’s postulates were met. One-way ANOVA was used to determine significant differences with a *P*-value of 0.05, supplemented by Duncan’s multiple range test to determine data significance. All data are presented as mean ± standard deviation.

## Results

### Phylogeny

**Figure 1:**
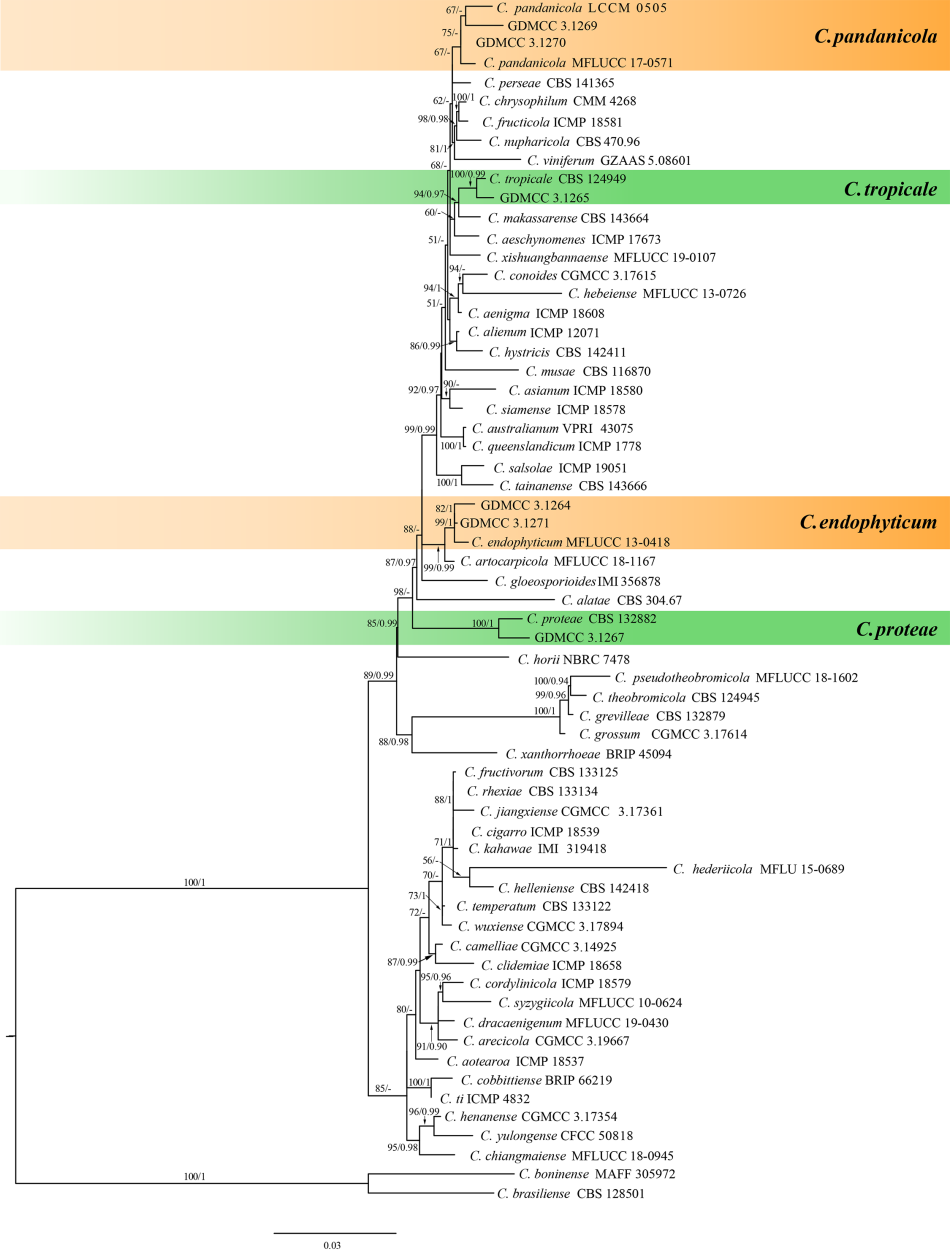
Maximum likelihood tree of *Colletotrichum gloeosporioides* species complex generated from combined ACT, CHS-1, GAPDH, ITS and TUB2 sequence data. Bootstrap support values ≥ 50% and Bayesian posterior probabilities ≥ 0.90 are demonstrated at the branches.

The combined sequence data set of *C. gloeosporioides* species complex comprised 1,905 characters (268 for ACT, 298 for CHS-1, 224 for GAPDH, 554 for ITS, and 495 for TUB2), including *C. boninense* (MAFF 305972) and *C. brasiliense* (CBS 128501) as the outgroup taxa. The best maximum likelihood (ML) tree revealed by RAxML is shown as a phylogram in Fig. 1. The topologies resulting from ML and Bayesian inference (BI) analyses of the concatenated data set were congruent. Isolates formed four individual clades representing, namely, *C. endophyticum, C. pandanicola, C. proteae and C. tropicale.*

**Figure 2:**
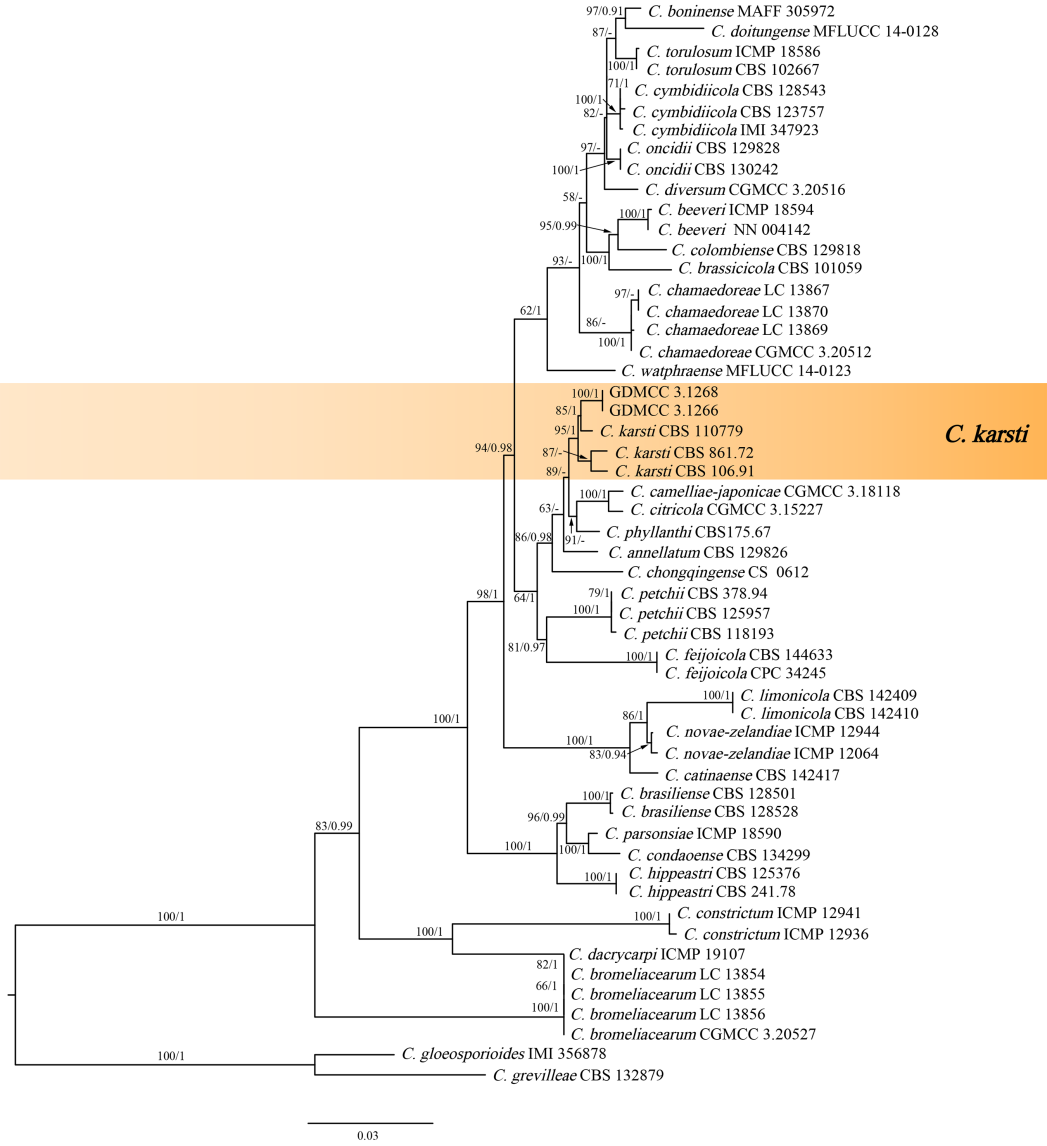
Maximum likelihood tree of *Colletotrichum boninense* species complex generated from combined ACT, CHS-1, GAPDH, ITS and TUB2 sequence data. Bootstrap support values ≥ 50% and Bayesian posterior probabilities ≥ 0.90 are demonstrated at the branches.

The combined sequence data set of *C. boninense* species complex comprised 1,839 characters (253 for ACT, 299 for CHS-1, 277 for GAPDH, 593 for ITS, and 483 for TUB2), with *C. gloeosporioides* (IMI 356878) and *C. grevilleae* (CPC 15481) as the outgroup taxa. The best maximum likelihood (ML) tree revealed by RAxML is shown as a phylogram in Fig. 2. The topologies resulting from ML and Bayesian inference (BI) analyses of the concatenated data set were congruent. Within the phylogeny of the *C. boninense* species complex (Fig. 2), our isolates clustered into a separate, strongly supported clade, representing the species *C. karsti.*

### Pathogenicity variation across mangrove hosts

**Figure 3:**
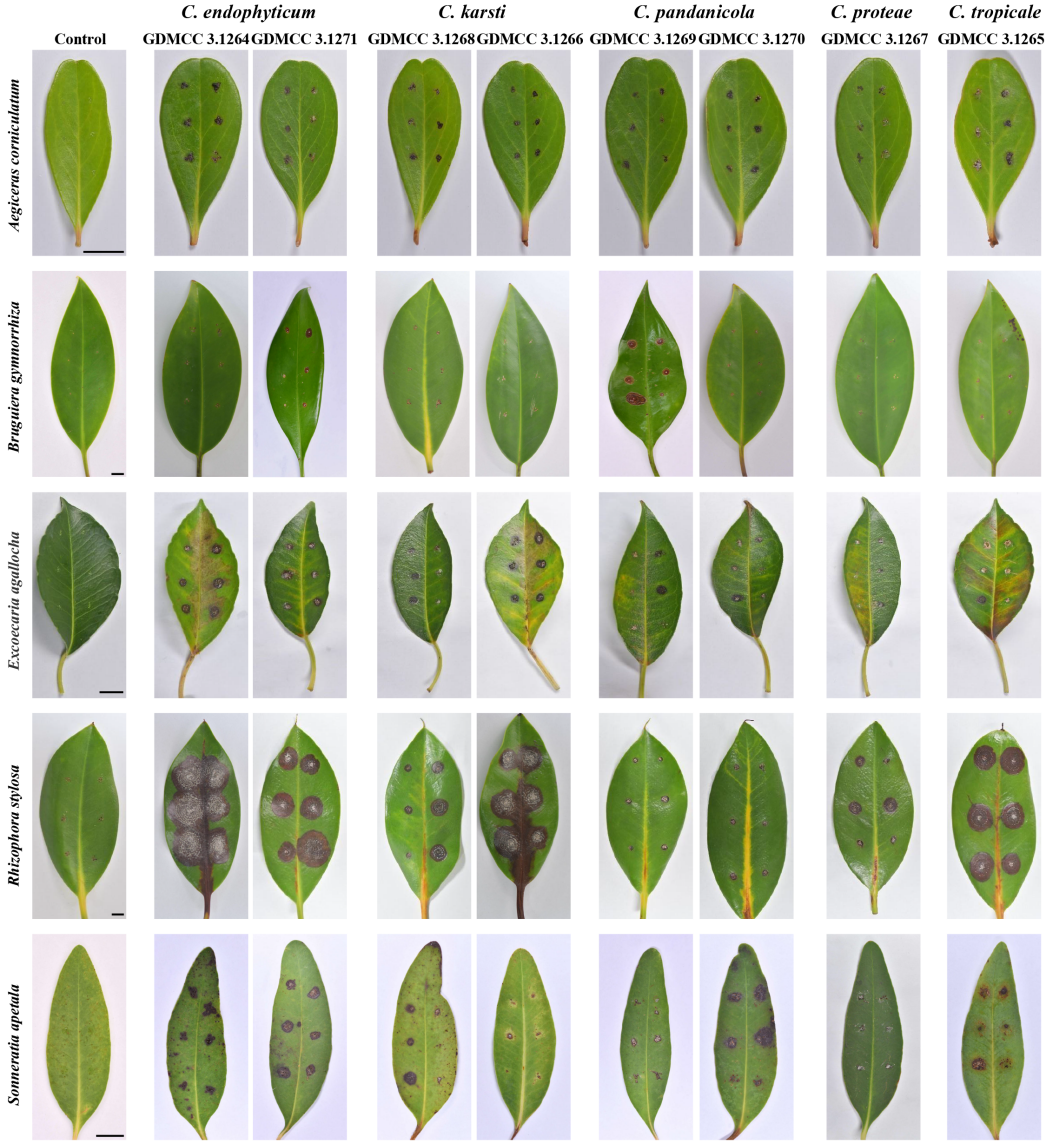
Pathogenicity test of the present * Colletotrichum* species on mangrove plants. Scale bar = 1 cm.

**Figure 4:**
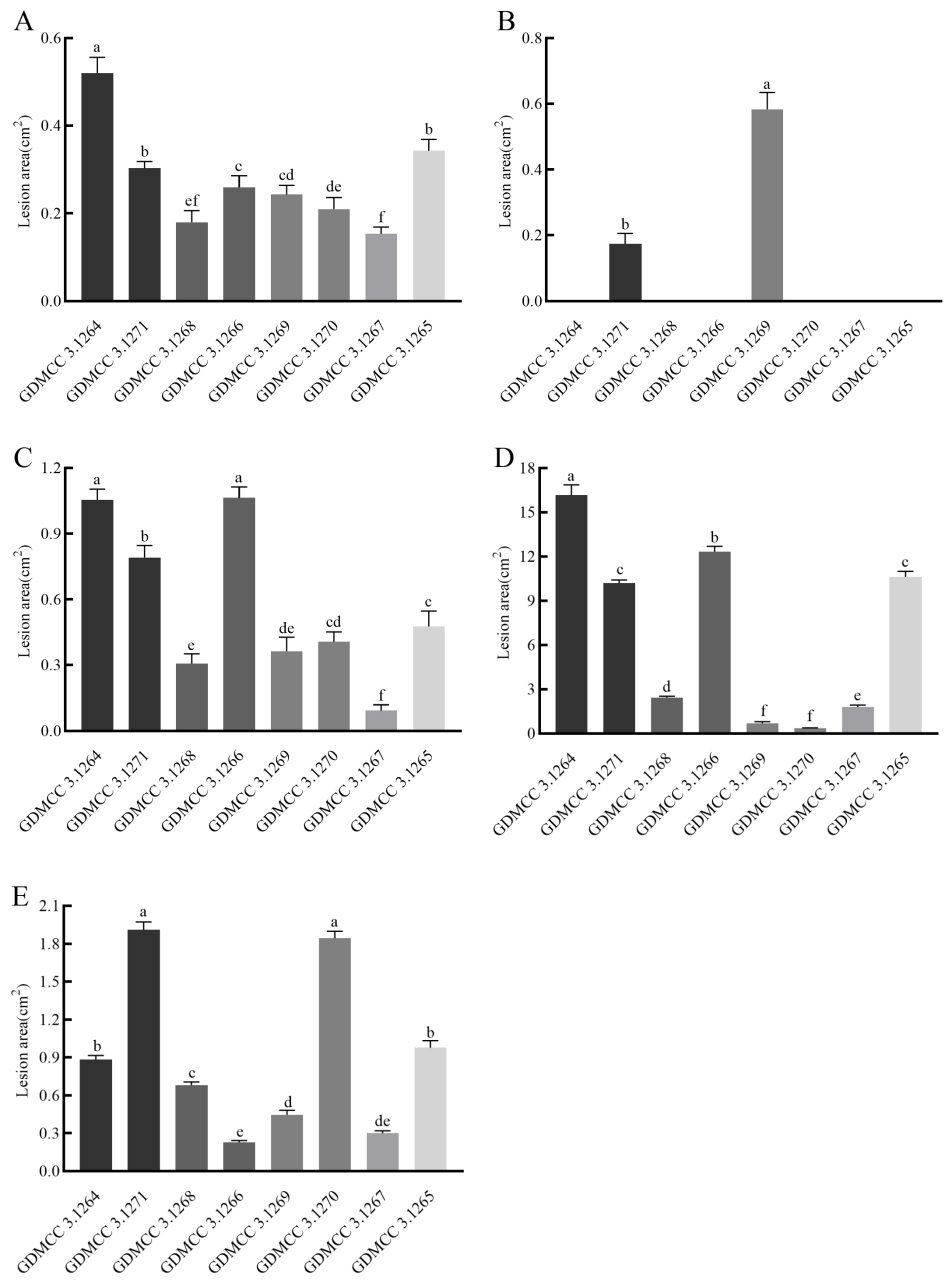
Lesion size on mangrove plants of * Colletotrichum* species in the ten days after inoculation. (A) Pathogenicity on *Aegiceras corniculatum*; (B) pathogenicity on *Bruguiera gymnorrhiza*; (C) pathogenicity on * Excoecaria agallocha*; (D) pathogenicity on *Rhizophora stylosa*; (E) pathogenicity on *Sonneratia apetala*. The lowercase letters of each treatment group are expressed as significant differences (*p* < 0.05).

Pathogenicity assays revealed significant interspecific and intraspecific virulence differences among five *Colletotrichum* species on five mangrove hosts, quantified by lesion area (Table S2). *Rhizophora stylosa* was highly susceptible to these *Colletotrichum* species in this study (Figs. 3 and 4). *Colletotrichum endophyticum* GDMCC 3.1264 was of the highest virulence with lesion area measuring 16.17 ± 0.69 cm^2^. Three strains *C*. *karsti* GDMCC 3.1266 (12.34 ± 0.35 cm^2^), *C. tropicale* GDMCC 3.1265 (10.62 ± 0.38 cm^2^), and *C. endophyticum* GDMCC 3.1271 (10.21 ± 0.20 cm^2^) exhibited high virulence. In contrast, *C. pandanicola* strains GDMCC 3.1269 and GDMCC 3.1270 showed significantly lower virulence on this host compared to the above fungal species. On *Excoecaria agallocha*, only *C. endophyticum* GDMCC 3.1264 and *C. karsti* GDMCC 3.1266 induced moderate lesions of 1–1.5 cm^2^ in lesion area. On *Sonneratia apetala*, *C. endophyticum* GDMCC 3.1271 (1.91 ± 0.23 cm^2^) and *C.pandanicola* GDMCC 3.1270 (1.84 ± 0.19 cm^2^) induced moderate lesions. On *Bruguiera gymnorrhiza*, only *C. pandanicola* GDMCC 3.1269 and *C. endophyticum* GDMCC 3.1271 demonstrated the ability to infect inducing small but distinct lesion areas of 0.58 ± 0.05 cm^2^ and 0.17 ± 0.03 cm^2^, respectively. On *Aegiceras corniculatum*, all pathogens induced only small lesion areas (≤ 0.52 ± 0.04 cm^2^) with no significant differences. Re-isolated pathogens from inoculated leaves matched original isolates in morphology and ITS sequences (NCBI BLASTn). These findings demonstrated host-specific interactions between the five mangrove species and *Colletotrichum* pathogens. *Rhizophora stylosa* exhibited broad susceptibility to multiple species, whereas *Bruguiera gymnorrhiza* showed distinct strain-specific susceptibility. Collectively, clear host-pathogen specificity governs *Colletotrichum*-mangrove interactions, with virulence spectrum ranging from strain-specific (*e.g.*, *Bruguiera gymnorrhiza*) to multi-susceptible (*e.g.*, *Rhizophora stylosa*).

### Taxonomy

#### *Colletotrichum endophyticum* Manamgoda et al. *Fungal Diversity* 61:110 2013

**Figure 5:**
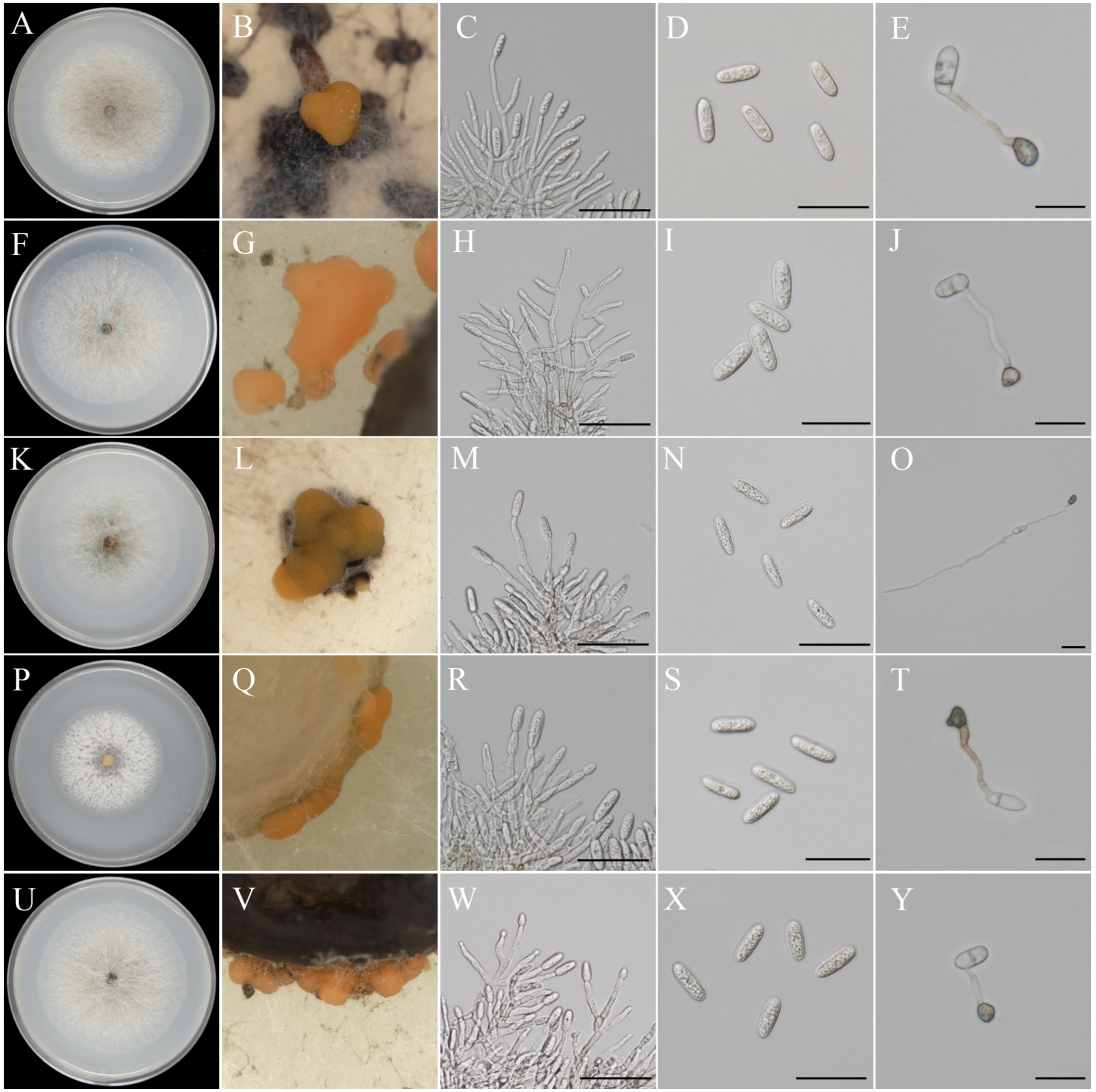
Morphological observation. (A, F, K, P, U) Colony morphology; (B, G, L, Q, V) conidial mass; (C, H, M, R, W) conidiophores and conidiogenous cells; (D, I, N, S, X) conidia; (E, J, O, T, Y) conidial gemination and appressorium; Scale bar = 20 µm.

Strains GDMCC 3.1264 and GDMCC 3.1271 were assigned as the first morphotype. Colonies were dense, cottony and milky, reaching diameters of 70–75 mm in 7 days incubated at 25 °C on OA (Fig. 5A), with orange conidial masses visible (Fig. 5B). Conidiophores were hyaline, smooth-walled, septate, branched, and ranged from cylindrical to inflated in shape (Fig. 5C). Conidia were hyaline, elongate-elliptical with blunt ends, measuring (13.2–16.3) µm × (4.8–6.3) µm (mean = 14.6 µm × 5.6 µm, *n* = 50) (Fig. 5D). Appressoria were light to dark brown, angular to irregular in shape, and measured (7.2–8.9) µm × (5.1–6.7) µm (mean = 8.1 µm × 5.9 µm, *n* = 20) (Fig. 5E). The isolates were phylogenetically closely related to *C. endophyticum* in the *C. gloeosporioides* species complex, forming a strongly supported lineage (BS = 99%) (Fig. 1). The ex-type culture has the following nucleotide similarities with the sequences of the ex-type of *C. endophyticum*. On ACT: 186/190 (97.89%), 207/208 (99.53%), respectively. On CHS-1: 278/279 (99.64%), 278/279 (99.64%), respectively. On GAPDH: 232/237 (97.89%), 208/213 (97.65%), respectively. On ITS: 446/449 (99.33%) and 458/458 (100%), respectively. On TUB: 669/670 (99.85%), 652/659 (98.94%). *Colletotrichum endophyticum* has been reported from *Ficus carica* and *Pennisetum purpureum* (Wang et al., 2023b; Manamgoda et al., 2013). The morphological characteristics of our collections are consistent with previous descriptions (Manamgoda et al., 2013). In the present study, this species was identified on *Bruguiera gymnorrhiza* and *Sonneratia apetala*, China, marking its first documented occurrence on this host.

#### *Colletotrichum karsti* Yang et al. *Cryptogamie*, *Mycologie*. 32(3):241 2011

Strains GDMCC 3.1266 and GDMCC 3.1268 belonged to the second morphotype. Colonies were white with orange-yellow pigmentation on OA, reaching diameters of 75–80 mm in 7 days (Fig. 5F). Orange conidial masses were observed (Fig. 5G). Conidiophores exhibited hyaline, septation, branching, and ranged from clavate to cylindrical in form (Fig. 5H). Conidia were elongate-elliptical, hyaline, and measured (12.9–17.6) µm × (4.9–7.0) µm (mean = 15.8 µm ×6.0 µm, *n* = 50) (Fig. 5I). Appressoria were dark brown, angular to irregular, and measured (6.7–8.2) µm × (4.5–5.7) µm (mean = 7.5 µm × 5.1 µm, *n* = 20) (Fig. 5J). These morphological characteristics were consistent with *C. karsti* (Sousa et al., 2021), confirming the taxonomic assignment. The isolates were phylogenetically closely related to *C. karsti* in the *C. boninense* species complex, forming a strongly supported lineage (BS >80%) (Fig. 2). The ex-type culture has the following nucleotide similarities with the sequences of the ex-type of *C. karsti*. On ACT: 188/199 (94.47%), 188/199 (94.47%), respectively. On CHS-1: 246/247 (99.60%), 245/247 (99.17%), respectively. On GAPDH: 203/207 (98.07%), 204/207 (98.55%), respectively. On ITS: 468/469 (99.79%) and 468/469 (99.79%), respectively. On TUB: 666/667 (99.85%), 677/679 (99.71%). *Colletotrichum karsti* has been reported from *Ginkgo*, *Rosa chinensis* and *Spigelia anthelmia* (Wang et al., 2025b; Norphanphoun et al., 2025; Sousa et al., 2021). The morphological characteristics of our collections are consistent with previous descriptions (Yang et al., 2011). In the present study, this species was identified on *Bruguiera gymnorrhiza* and *Sonneratia apetala*, China, marking its first documented occurrence on this host.

#### *Colletotrichum pandanicola* Tibpromma et al. *MycoKeys* 33:47 2018

Strains GDMCC 3.1269 and GDMCC 3.1270 belonged to the third morphotype. Colonies on OA flat with entire edge, gray to white, with sparse to moderate aerial mycelium, reaching diameters of 80–85 mm in 7 days incubated at 25 °C on OA (Fig. 5K). Orange conidial masses were observed (Fig. 5L). Conidiophores were hyaline, septate, branched, and cylindrical to clavate in shape (Fig. 5M). Conidia were oblong and hyaline with bluntly rounded apices, measuring (11.6–15.2) µm × (4.3–6.1) µm (mean = 13.7  × 4.8 µm, *n* = 50) (Fig. 5N). Appressoria were light to dark brown, angular to irregular in shape, with entire margins, measuring (6.9–8.5) µm × (4.7–5.6) µm (mean = 7.5 µm ×5.2 µm, *n* = 20) (Fig. 5O). The isolates GDMCC 3.1269 and GDMCC 3.1270 were phylogenetically closely related to *C. pandanicola* in the *C. gloeosporioides* species complex, forming a strongly supported lineage (BS >60%) (Fig. 1). The ex-type culture has the following nucleotide similarities with the sequences of the ex-type of *C. pandanicola*. On ACT: 181/185 (97.84%), 185/188 (98.40%), respectively. On CHS-1: 275/280 (98.21%), 278/280 (99.29%), respectively. On GAPDH: 204/211 (96.68%), 247/248 (99.60%), respectively. On ITS: 449/450 (99.78%) and 450/450 (100%), respectively. On TUB: 479/480 (99.79%), 480/480 (100%). *Colletotrichum pandanicola* has been reported from the China and Thailand, where it occurs on *Strawberry* and *Pandanaceae* (Tibpromma et al., 2018; Yan et al., 2022). The morphological characteristics of our collections are consistent with previous descriptions (Tibpromma et al., 2018; Yan et al., 2022). In the present study, this species was identified on *Bruguiera gymnorrhiza*, China, marking its first documented occurrence on this host.

#### *Colletotrichum proteae* Liu et al. *Fungal Diversity* 61:100 2013

Strain GDMCC 3.1267 represented the fourth morphotype. Colonies were white on OA with irregular margins, reaching diameters of 70–75 mm in 7 days (Fig. 5P). Orange conidial masses were observed (Fig. 5Q). Conidiophores were hyaline, septate, branched, and ranged from cylindrical to inflate in shape (Fig. 5R). Conidia were long-elliptical, hyaline, and measured (15.2–20.6) µm × (5.0–6.3) µm (mean = 17.7 µm ×5.5 µm, *n* = 50) (Fig. 5S). Appressoria were dark brown, angular to irregular, and measured (7.5–9.2) µm × (4.7–5.9) µm (mean = 8.4 µm ×5.3 µm, *n* = 20) (Fig. 5T). The isolates were phylogenetically closely related to *C. proteae* in the *C. gloeosporioides* species complex, forming a strongly supported lineage (BS = 100%) (Fig. 1). The ex-type culture has the following nucleotide similarities with the sequences of the ex-type of *C. proteae*. On ACT: 185/189 (97.88%), respectively. On CHS-1: 299/301 (99.34%), respectively. On GAPDH: 210/222 (94.59%), respectively. On ITS: 452/456 (99.12%), respectively. On TUB: 659/664 (99.25%). *Colletotrichum proteae* has been reported exclusively on hosts of the *Proteaceae* (Liu et al., 2013). The morphological characteristics of our collections are consistent with previous descriptions (Liu et al., 2013). In the present study, this species was identified on *Rhizophora stylosa*, China, marking its first documented occurrence on this host.

#### *Colletotrichum tropicale* Rojas et al. *Mycologia* 102(6):1331 2010

Strain GDMCC 3.1265 belonged to the fifth morphotype. Colonies were dense and milky white, reaching diameters of 70–75 mm in 7 days incubated at 25 °C on OA (Fig. 5U). Orange conidial produced on OA (Fig. 5V). Conidiophores were hyaline, septate, branched, and cylindrical to clavate in shape (Fig. 5W). Conidia were hyaline, elongate-elliptical, with bluntly rounded apices and slight basal tapering, measuring (12.6–12.7) µm × (4.0–5.4) µm (mean = 14.4 µm × 4.8 µm, *n* = 50) (Fig. 5X). Appressoria were angular to irregular, light to dark gray in color, and measured (7.4–8.6) µm × (5.2–6.7) µm (mean = 8.0 µm × 5.9 µm, *n* = 20) (Fig. 5Y). The isolates were phylogenetically closely related to *C. tropicale* in the *C. gloeosporioides* species complex, forming a strongly supported lineage (BS = 100%) (Fig. 1). The ex-type culture has the following nucleotide similarities with the sequences of the ex-type of *C. tropicale*. On ACT: 179/179 (100%), respectively. On CHS-1: 240/244 (98.36%), respectively. On GAPDH: 207/212 (97.64%), respectively. On ITS: 446/449 (99.33%), respectively. On TUB: 665/665 (100%). *Colletotrichum tropicale* has been reported from *Butyrospermum parkii* and *Mangifera indica* (Yan et al., 2022; Zhao et al., 2022). The morphological characteristics of our collections are consistent with previous descriptions (Rojas et al., 2010). In the present study, this species was identified on *Excoecaria agallocha*, China, marking its first documented occurrence on this host.

## Discussion

Accurate species identification is essential for disease control, and taxonomic classification within the genus *Colletotrichum* remains a significant research focus (Dean et al., 2012). Early classification of *Colletotrichum* was primarily based on morphological traits and host associations (Yang et al., 2011). However, taxonomic resolution within this genus has long been hindered by insufficient morphological differentiation for species delimitation and the lack of strict host specificity in many *Colletotrichum* species (Cai et al., 2009). With advancements in molecular techniques (White et al., 1990), an increasing number of molecular markers have been employed in *Colletotrichum* taxonomy (Bhunjun et al., 2021). Currently, *Colletotrichum* is classified into 16 species complexes and over 300 species based on integrated morphological and multilocus phylogenetic analyses (ACT, CHS-1, GAPDH, ITS, and TUB2) (Liu et al., 2022). In this study, five *Colletotrichum* species associated with mangrove anthracnose were identified based on integrated morphological and phylogenetic analyses: *C. endophyticum*, *C. karsti*, *C. pandanicola*, *C. proteae* and *C. tropicale*. *Colletotrichum gloeosporioides* is the species complex that includes many morphologically similar pathogens of anthracnose (Weir, Johnston & Damm, 2012). Among these, *C. endophyticum*, *C. pandanicola*, *C. proteae* and *C. tropicale* belong to the *C. gloeosporioides* species complex (Li et al., 2019; Jayawardena et al., 2016). To date, no prior reports have documented the occurrence of *C. endophyticum*, *C. karsti*, *C. pandanicola*, *C. proteae* and *C. tropicale* in mangrove hosts including *Aegiceras corniculatum*, *Bruguiera gymnorrhiza*, *Excoecaria agallocha*, *Rhizophora stylosa* and *Sonneratia apetala*. This study provides the first record of these five pathogenic fungi in mangrove anthracnose and addresses a critical gap in the current taxonomy of mangrove fungal pathogens.

*Colletotrichum* is a cosmopolitan genus of plant pathogens known for its broad host range and ability to infect both monocot and dicot species (Meng et al., 2019). The five species central to this study—*Colletotrichum endophyticum*, *C. karsti*, *C. pandanicola*, *C. proteae* and *C. tropicale*—serve as specific examples of this ecological adaptability. Their ability to infect a range of hosts, as observed in our experiments, aligns with the general trend in the genus where a single species is able to infect multiple hosts, and one host can be affected by multiple species. Existing research indicates that *C. fructicola* and *C. siamense* are both recognized as pathogens possessing broad host ranges in the *Colletotrichum* genus. *Colletotrichum fructicola* has been reported to infect numerous hosts, including *Cunninghamia lanceolata* (Han et al., 2021; Jiang et al., 2025), *Camellia oleifera* (Chen et al., 2024), and *Polygonatum cyrtonema* (Dan et al., 2023). Furthermore, *C. siamense* has been shown to be capable of infecting more than 60 plant species spanning various families (Ji et al., 2018), including causes canker on *Quercus* L. (Wang et al., 2023a) and infects *Machilus pauhoi* (Lv et al., 2023). *Colletotrichum aenigma* causes anthracnose in strawberry (Hou et al., 2022), *Dioscorea alata* L. (Han, 2012), and other crops. *Colletotrichum karsti* has been isolated from *Cymbidium* spp. (Yang et al., 2011), peppers (Tozze et al., 2009), apples (Velho et al., 2014) and Tea-Oil Trees (Jiang & Li, 2018). *Colletotrichum acutatum* and *C. gloeosporioides* cause anthracnose on *Canarium album* (Talhinhas et al., 2015). Meanwhile, *C. fructicola*, *C. tropicale*, and *C. fioriniae* infect *Liquidambar formosana* (Zhu et al., 2019). The pathogens identified in this study exhibited no host specificity.

The five tested mangrove plants exhibited differential host susceptibility to *Colletotrichum* strains. *Rhizophora stylosa* emerged as a highly susceptible host, exhibiting extensive lesions when inoculated with multiple *Colletotrichum* species (*C. endophyticum*, *C. karsti* and *C. tropicale*). Only two of eight strains (*C*. *pandanicola* GDMCC 3.1269 and *C*. *endophyticum* GDMCC 3.1271) induced limited lesions on *Bruguiera gymnorrhiza*, which indicating *Bruguiera gymnorrhiza* was genetic incompatibility with most pathogens, demonstrated its strain-specific resistance. *Sonneratia apetala* and *Aegiceras corniculatum* showed non-host resistance, with consistently small lesions (<0.52 cm^2^) across all strains (*P* > 0.05). Moreover, intraspecific virulence variation in these tested strains pathogenicity hinted diverged significantly within species. The two *C. endophyticum* and two *C*. *karsti* strains exhibited different virulence inducing distinct lesion development on most tested host plants. While only one strain of *C. endophyticum* and *C*. *pandanicola* were among the few pathogens capable of infecting *Bruguiera gymnorrhiza*, thus demonstrating that virulence is strain-dependent rather than species-level trait (Veloso et al., 2021).

Several methodological limitations should be considered when interpreting the results of this study. Although the wounded detached-leaf assay is widely used for initial pathogenicity screening of *Colletotrichum* species (Andrivon et al., 1998; De Silva et al., 2017; Song et al., 2021), wounding bypasses natural physical barriers such as the cuticle and epidermis. Consequently, this method primarily assesses the capacity for wound colonization rather than the ability to infect through natural routes (*e.g.*, direct penetration). Moreover, the absence of *Colletotrichum* isolates from non-mangrove hosts prevents distinguishing true host specificity from general wound susceptibility. While our results reveal differential virulence under controlled conditions, studies using intact plants with non-wound inoculation, cross-host pathogen comparisons, and field-validated host-jumps assays are essential to confirm ecological relevance.

In summary, mangrove anthracnose was identified using both morphological and molecular diagnostic approaches, and preliminary virulence assessments were conducted. However, further research is required to elucidate epidemiological patterns and mechanisms of infection. These results could offer a theoretical basis for resistance breeding and germplasm screening to enhance anthracnose control in mangrove ecosystems.

## Conclusion

Five pathogenic fungi, including *C.endophyticum, C. karsti, C. pandanicola, C. proteae and C. tropicale*, were identified in this study as the causal agents of anthracnose in five mangrove plant species, resulting in the formation of leaf spots. This study enhances our understanding of the etiology of mangrove anthracnose and offers foundational insights to support the development and implementation of effective control strategies for future management efforts.

##  Supplemental Information

10.7717/peerj.21307/supp-1 Supplemental Information 1 Taxa used in this study for the analysis of combined ACT, CHS-1, GAPDH, ITS , and TUB2 sequence data and their GenBank accession numbers. The ex-type strains are indicated with asterisk (*). Bold blue indicated the sequences generated in this Note: BRIP: The Building Respect for Intellectual Property Database Project. CBS: Centraalbureau voor Schimmelcultures. CFCC: China Forest Certification Council. CGMCC: China General Microbiological Culture Collection Center. CMM: Culture Collection of Phytopathogenic Fungi “Prof. Maria Menezes”, Universidade Federal Ruralde Pernambuco, Recife, Brazil . CPC: Cooperative Patent Classification. CS: Australian National Algae Culture Collection, ANACC Castray Esplanade, Hobart, Tasmania. GDMCC: Guangdong Microbial Culture Collection. GZAAS: Guizhou Academy of Agricultural Sciences. ICMP: International Collection of Microorganisms from Plants. IMI: International Mycological Institute. LC: LC Culture Collection (a personal culture collection of Lei Cai, housed in the Institute of Microbiology, Chinese Academy of Sciences). MAFF: Ministry of Agriculture, Forestry and Fisheries. MFLUCC: Mae Fah Luang University Culture Collection, Chiang Rai, Thailand. NBRC: NITE Biological Resource Center. NN: The CB Rhizobium Collection, South Penrith Distribution Centre. VPRI: Victorian Plant Pathology Herbarium.

10.7717/peerj.21307/supp-2 Supplemental Information 2 Pathogenicity of fungal on different hosts Note: The data are presented as mean ± standard deviation, and the lowercase letters of each treatment group are expressed as significant differences (*p* < 0.05).

10.7717/peerj.21307/supp-3 Supplemental Information 3 Original data of TUB2 sequence

10.7717/peerj.21307/supp-4 Supplemental Information 4 Original data of CHS-1 sequence

10.7717/peerj.21307/supp-5 Supplemental Information 5 Original data of GAPDH sequence

10.7717/peerj.21307/supp-6 Supplemental Information 6 The pathogenic area of the strains to different hosts

## Additional Information and Declarations

### Data Availability

The following information was supplied regarding data availability: The data is available in the Supplemental Files. The ACT sequences are accessible via GenBank: PX229581, PX229587, PX229583, PX229586, PX229584, PX229585, PX206619 and PX229582. The CHS-1 sequences are accessible *via* GenBank: PX436189, PX436195, PX436191, PX436194, PX436192, PX436193, PX436188 to PX436190. The GAPDH sequences are accessible *via* GenBank: PX654197, PX654191, PX654195, PX654192, PX654194, PX654193, PX654198 and PX654196. The ITS sequences are accessible *via* GenBank: PX024417, PX024423, PX024419, PX024422, PX024420, PX024421, PX024416 and PX024418. The TUB2 sequences are accessible via GenBank: PX365742, PX654186, PX654190, PX654187, PX654189, PX654188, PX257449 and PX501982.
