# Supplementary material for: Occurrence and identification of Colletotrichum species in mangrove-associated anthracnose
Source: PeerJ. 2026 Jun 3;14:e21307. doi: 10.7717/peerj.21307 (PMC13242191; doi:10.7717/peerj.21307)
Supplement: Supplemental Information 1 — Note: BRIP: The Building Respect for Intellectual Property Database Project. CBS: Centraalbureau voor Schimmelcultures. CFCC: China Forest Certification Council. CGMCC: China General Microbiological Culture Collection Center. CMM: Culture Collection of Phytopathogenic Fungi “Prof. Maria Menezes”, Universidade Federal Ruralde Pernambuco, Recife, Brazil . CPC: Cooperative Patent Classification. CS: Australian National Algae Culture Collection, ANACC Castray Esplanade, Hobart, Tasmania. GDMCC: Guangdong Microbial Culture Collection. GZAAS: Guizhou Academy of Agricultural Sciences. ICMP: International Collection of Microorganisms from Plants. IMI: International Mycological Institute. LC: LC Culture Collection (a personal culture collection of Lei Cai, housed in the Institute of Microbiology, Chinese Academy of Sciences). MAFF: Ministry of Agriculture, Forestry and Fisheries. MFLUCC: Mae Fah Luang University Culture Collection, Chiang Rai, Thailand. NBRC: NITE Biological Resource Center. NN: The CB Rhizobium Collection, South Penrith Distribution Centre. VPRI: Victorian Plant Pathology Herbarium. [file peerj-14-21307-s001.docx]

**Table S1 |** Taxa used in this study for the analysis of combined ACT, CHS-1, GAPDH, ITS, and TUB2 sequence data and their GenBank accession numbers. The ex-type strains are indicated with asterisk (*). Bold blue indicated the sequences generated in this study.

| **Species** | **Culture** | **GenBank accession number** | | | | |
| --- | --- | --- | --- | --- | --- | --- |
|  |  | **ACT** | **CHS-1** | **GAPDH** | **ITS** | **TUB2** |
| *Colletotrichum aenigma* | ICMP 18608* | JX009443 | JX009774 | JX010044 | JX010244 | JX010389 |
| *C. aeschynomenes* | ICMP 17673* | JX009483 | JX009799 | JX009930 | JX010176 | JX010392 |
| *C. alatae* | CBS 304.67* | JX009471 | JX009837 | JX009990 | JX010190 | JX010383 |
| *C. alienum* | ICMP 12071* | JX009572 | JX009882 | JX010028 | JX010251 | JX010411 |
| *C. aotearoa* | ICMP 18537* | JX009564 | JX009853 | JX010005 | JX010205 | JX010420 |
| *C. arecicola* | CGMCC 3.19667* | MK935374 | MK935541 | MK935455 | MK914635 | MK935498 |
| *C. artocarpicola* | MFLUCC 18-1167* | MN435570 | MN435569 | MN435568 | MN415991 | MN435567 |
| *C. asianum* | ICMP 18580* | JX009584 | JX009867 | JX010053 | FJ972612 | JX010406 |
| *C. australianum* | VPRI 43075* | MN442109 | MW091987 | MG572127 | MG572138 | MG572149 |
| *C. camelliae* | CGMCC 3.14925* | KJ954363 | MZ799255 | KJ954782 | KJ955081 | KJ955230 |
| *C. pandanicola* | LCCM 0505 | OM869431 | OM869430 | OM869429 | OM841534 | OM869433 |
| *C. chiangmaiense* | MFLUCC 18-0945* | MW655578 | MW623653 | MW548592 | MW346499 | - |
| *C. chrysophilum* | CMM 4268* | KX093982 | KX094083 | KX094183 | KX094252 | KX094285 |
| *C. cigarro* | ICMP 18539* | JX009523 | JX009800 | JX009966 | JX010230 | JX010434 |
| *C. clidemiae* | ICMP 18658* | JX009537 | JX009877 | JX009989 | JX010265 | JX010438 |
| *C. cobbittiense* | BRIP 66219* | MH094134 | MH094135 | MH094133 | MH087016 | MH094137 |
| *C. conoides* | CGMCC 3.17615* | KP890144 | KP890156 | KP890162 | KP890168 | KP890174 |
| *C. cordylinicola* | ICMP 18579* | HM470235 | JX009864 | JX009975 | JX010226 | JX010440 |
| *C. dracaenigenum* | MFLUCC 19-0430* | MT313686 | MT215575 | MT215577 | MN921250 | - |
| *C. endophyticum* | MFLUCC 13-0418 | KF306258 | MZ799261 | KC832854 | KC633854 | MZ673954 |
| *C. fructicola* | ICMP 18581* | FJ907426 | JX009866 | JX010033 | JX010165 | JX010405 |
| *C. fructivorum* | CBS 133125* | MZ664126 | MZ799259 | MZ664047 | JX145145 | JX145196 |
| *C. gloeosporioides* | IMI 356878* | JX009531 | JX009818 | JX010056 | JX010152 | JX010445 |
| *C. grevilleae* | CBS 132879* | KC296941 | KC296987 | KC297010 | KC297078 | KC297102 |
| *C. grossum* | CGMCC 3.17614* | KP890141 | KP890153 | KP890159 | KP890165 | KP890171 |
| *C. hebeiense* | MFLUCC 13–0726* | KF377532 | KF289008 | KF377495 | KF156863 | KF288975 |
| *C. hederiicola* | MFLU 15-0689* | MN635795 | MN635794 | - | MN631384 | - |
| *C. helleniense* | CBS 142418* | KY856019 | KY856186 | KY856270 | KY856446 | KY856528 |
| *C. henanense* | CGMCC 3.17354* | KM023257 | MZ799256 | KJ954810 | KJ955109 | KJ955257 |

(Continues)

**Table S1 |** (Continues)

| **Species** | **Culture** | **GeneBank assession number** | | | | |
| --- | --- | --- | --- | --- | --- | --- |
|  |  | **ACT** | **CHS-1** | **GAPDH** | **ITS** | **TUB2** |
| *C. horii* | NBRC 7478* | JX009438 | JX009752 | GQ329681 | GQ329690 | JX010450 |
| *C. hystricis* | CBS 142411* | KY856023 | KY856190 | KY856274 | KY856450 | KY856532 |
| *C. jiangxiense* | CGMCC 3.17361* | KJ954427 | MZ799257 | KJ954850 | KJ955149 | OK236389 |
| *C. kahawae* | IMI 319418* | JX009452 | JX009813 | JX010012 | JX010231 | JX010444 |
| *C. makassarense* | CBS 143664* | MH781480 | MH805850 | MH728820 | MH728812 | MH846563 |
| *C. musae* | CBS 116870* | JX009433 | JX009896 | JX010050 | JX010146 | HQ596280 |
| *C. nupharicola* | CBS 470.96* | JX009437 | JX009835 | JX009972 | JX010187 | JX010398 |
| *C. pandanicola* | MFLUCC 17-0571* | MG646938 | MG646931 | MG646934 | MG646967 | MG646926 |
| *C. perseae* | CBS 141365* | KX620145 | MZ799260 | KX620242 | KX620308 | KX620341 |
| *C. proteae* | CBS 132882* | KC296940 | KC296986 | KC297009 | KC297079 | KC297101 |
| *C. pseudotheobromicola* | MFLUCC 18–1602* | MH853681 | MH853678 | MH853675 | MH817395 | MH853684 |
| *C. queenslandicum* | ICMP 1778* | JX009447 | JX009899 | JX009934 | JX010276 | JX010414 |
| *C. rhexiae* | CBS 133134* | MZ664127 | MZ799258 | MZ664046 | JX145128 | JX145179 |
| *C. salsolae* | ICMP 19051* | JX009562 | JX009863 | JX009916 | JX010242 | JX010403 |
| *C. siamense* | ICMP 18578* | FJ907423 | JX009865 | JX009924 | JX010171 | JX010404 |
| *C. syzygiicola* | MFLUCC 10-0624* | KF157801 | - | KF242156 | KF242094 | KF254880 |
| *C. tainanense* | CBS 143666* | MH781475 | MH805845 | MH728823 | MH728818 | MH846558 |
| *C. temperatum* | CBS 133122* | MZ664125 | MZ799254 | MZ664045 | JX145159 | JX145211 |
| *C. theobromicola* | CBS 124945* | JX009444 | JX009869 | JX010006 | JX010294 | JX010447 |
| *C. ti* | ICMP 4832* | JX009520 | JX009898 | JX009952 | JX010269 | JX010442 |
| *C. tropicale* | CBS 124949* | JX009489 | JX009870 | JX010007 | JX010264 | JX010407 |
| *C. viniferum* | GZAAS 5.08601* | JN412795 | - | JN412798 | JN412804 | - |
| *C. wuxiense* | CGMCC 3.17894* | KU251672 | KU251939 | KU252045 | KU251591 | KU252200 |
| *C. xanthorrhoeae* | BRIP 45094* | JX009478 | JX009823 | JX009927 | JX010261 | JX010448 |
| *C. xishuangbannaense* | MFLUCC 19-0107* | MW652294 | MW660832 | MW537586 | MW346469 | - |

(Continues)

**Table S1 |** (Continues)

| **Species** | **Culture** | **GeneBank assession number** | | | | |
| --- | --- | --- | --- | --- | --- | --- |
|  |  | **ACT** | **CHS-1** | **GAPDH** | **ITS** | **TUB2** |
| *C. yulongense* | CFCC 50818* | MH777394 | MH793605 | MK108986 | MH751507 | MK108987 |
| *C. boninense* | MAFF 305972* | JQ005501 | JQ005327 | JQ005240 | JQ005153 | JQ005588 |
| C. brasiliense | CBS 128501 | JQ005583 | JQ005409 | JQ005322 | JQ005235 | JQ005669 |
| *C. annellatum* | CBS 129826 | JQ005570 | JQ005396 | JQ005309 | JQ005222 | JQ005656 |
| *C. beeveri* | ICMP 18594* | JQ005519 | JQ005345 | JQ005258 | JQ005171 | JQ005605 |
| *C. beeveri* | NN 004142 | MZ664179 | MZ799277 | MZ664082 | MZ595881 | - |
| *C. brasiliense* | CBS 128501 | JQ005583 | JQ005409 | JQ005322 | JQ005235 | JQ005669 |
| *C. brasiliense* | CBS 128528 | JQ005582 | JQ005408 | JQ005321 | JQ005234 | JQ005668 |
| *C. brassicicola* | CBS 101059 | JQ005520 | JQ005346 | JQ005259 | JQ005172 | JQ005606 |
| *C. bromeliacearum* | CGMCC 3.20527* | MZ664130 | MZ799267 | MZ664077 | MZ595832 | MZ673956 |
| *C. bromeliacearum* | LC 13854 | MZ664131 | MZ799268 | MZ664078 | MZ595833 | OK360930 |
| *C. bromeliacearum* | LC 13855 | MZ664132 | MZ799269 | MZ664079 | MZ595834 | OK360931 |
| *C. bromeliacearum* | LC 13856 | MZ664133 | MZ799270 | MZ664080 | MZ595835 | OK360932 |
| *C. camelliae–japonicae* | CGMCC 3.18118* | KX893576 | MZ799271 | KX893584 | KX853165 | KX893580 |
| *C. catinaense* | CBS 142417* | KY855971 | KY856136 | KY856224 | KY856400 | KY856482 |
| *C. chamaedoreae* | LC 13867 | MZ664187 | MZ799273 | MZ664083 | MZ595889 | MZ674007 |
| *C. chamaedoreae* | CGMCC 3.20512* | MZ664188 | MZ799274 | MZ664084 | MZ595890 | MZ674008 |
| *C. chamaedoreae* | LC 13869 | MZ664189 | MZ799275 | MZ664086 | MZ595891 | MZ674009 |
| *C. chamaedoreae* | LC 13870 | MZ664190 | MZ799276 | MZ664085 | MZ595892 | MZ674010 |
| *C. citricola* | CGMCC 3.15227* | KC293616 | - | KC293736 | KC293576 | KC293656 |
| *C. chongqingense* | CS 0612* | MT976107 | MT976117 | MG602022 | MG602060 | MG602044 |
| *C. colombiense* | CBS 129818* | JQ005522 | JQ005348 | JQ005261 | JQ005174 | JQ005608 |
| *C. condaoense* | CBS 134299* | - | MH229926 | MH229920 | MH229914 | MH229923 |
| *C. constrictum* | ICMP 12941* | JQ005586 | JQ005412 | JQ005325 | JQ005238 | JQ005672 |
| *C. constrictum* | ICMP 12936 | JQ005585 | JQ005411 | JQ005324 | JQ005237 | JQ005671 |
| *C. cymbidiicola* | IMI 347923* | JQ005514 | JQ005340 | JQ005253 | JQ005166 | JQ005600 |
| *C. cymbidiicola* | CBS 128543 | JQ005515 | JQ005341 | JQ005254 | JQ005167 | JQ005601 |

(Continues)

**Table S1 |** (Continues)

| **Species** | **Culture** | **GeneBank assession number** | | | | |
| --- | --- | --- | --- | --- | --- | --- |
|  |  | **ACT** | **CHS-1** | **GAPDH** | **ITS** | **TUB2** |
| *C. cymbidiicola* | CBS 123757 | JQ005516 | JQ005342 | JQ005255 | JQ005168 | JQ005602 |
| *C. dacrycarpi* | ICMP 19107* | JQ005584 | JQ005410 | JQ005323 | JQ005236 | JQ005670 |
| *C. diversum* | CGMCC 3.20516* | MZ664142 | MZ799272 | MZ664081 | MZ595844 | MZ673965 |
| *C. doitungense* | MFLUCC 14-0128* | MH376385 | - | MH049480 | MF448524 | MH351277 |
| *C. feijoicola* | CBS 144633* | MK876466 | - | MK876475 | MK876413 | MK876507 |
| *C. feijoicola* | CPC 34245 | MK876465 | MK876471 | MK876474 | MK876414 | MK876506 |
| *C. hippeastri* | CBS 125376* | JQ005579 | JQ005405 | JQ005318 | JQ005231 | JQ005665 |
| *C. hippeastri* | CBS 241.78 | JQ005580 | JQ005406 | JQ005319 | JQ005232 | JQ005666 |
| *C. karsti* | CBS 861.72 | JQ005532 | JQ005358 | JQ005271 | JQ005184 | JQ005618 |
| *C. karsti* | CBS 106.91 | JQ005568 | JQ005394 | JQ005307 | JQ005220 | JQ005654 |
| *C. karsti* | CBS 110779 | JQ005546 | JQ005372 | JQ005285 | JQ005198 | JQ005632 |
| *C. limonicola* | CBS 142409 | KY856044 | KY856212 | KY856295 | KY856471 | KY856553 |
| *C. limonicola* | CBS 142410* | KY856045 | KY856213 | KY856296 | KY856472 | KY856554 |
| *C. novae–zelandiae* | ICMP 12944* | JQ005576 | JQ005402 | JQ005315 | JQ005228 | JQ005662 |
| *C. novae–zelandiae* | ICMP 12064 | JQ005577 | JQ005403 | JQ005316 | JQ005229 | JQ005663 |
| *C. oncidii* | CBS 129828* | JQ005517 | JQ005343 | JQ005256 | JQ005169 | JQ005603 |
| *C. oncidii* | CBS 130242 | JQ005518 | JQ005344 | JQ005257 | JQ005170 | JQ005604 |
| *C. parsonsiae* | ICMP 18590* | JQ005581 | JQ005407 | JQ005320 | JQ005233 | JQ005667 |
| *C. petchii* | CBS 378.94* | JQ005571 | JQ005397 | JQ005310 | JQ005223 | JQ005657 |
| *C. petchii* | CBS 118193 | JQ005575 | JQ005401 | JQ005314 | JQ005227 | JQ005661 |
| *C. petchii* | CBS 125957 | JQ005574 | JQ005400 | JQ005313 | JQ005226 | JQ005660 |
| *C. phyllanthi* | CBS 175.67* | JQ005569 | JQ005395 | JQ005308 | JQ005221 | JQ005655 |
| *C. torulosum* | ICMP 18586* | JQ005512 | JQ005338 | JQ005251 | JQ005164 | JQ005598 |
| *C. torulosum* | CBS 102667 | JQ005513 | JQ005339 | JQ005252 | JQ005165 | JQ005599 |
| *C. watphraense* | MFLUCC 14-0123* | MH376384 | - | MH049479 | MF448523 | MH351276 |
| ***C. endophyticum*** | **GDMCC 3.1264** | **PX229581** | **PX436189** | **PX654197** | **PX024417** | **PX365742** |
| ***C. endophyticum*** | **GDMCC 3.1271** | **PX229587** | **PX436195** | **PX654191** | **PX024423** | **PX654186** |
| ***C. karsti*** | **GDMCC 3.1268** | **PX229583** | **PX436191** | **PX654195** | **PX024419** | **PX654190** |

(Continues)

**Table S1 |** (Continues)

| **Species** | **Culture** | **GeneBank assession number** | | | | |
| --- | --- | --- | --- | --- | --- | --- |
|  |  | **ACT** | **CHS-1** | **GAPDH** | **ITS** | **TUB2** |
| ***C. karsti*** | **GDMCC 3.1266** | **PX229586** | **PX436194** | **PX654192** | **PX024422** | **PX654187** |
| ***C. pandanicola*** | **GDMCC 3.1269** | **PX229584** | **PX436192** | **PX654194** | **PX024420** | **PX654189** |
| ***C. pandanicola*** | **GDMCC 3.1270** | **PX229585** | **PX436193** | **PX654193** | **PX024421** | **PX654188** |
| ***C*. *proteae*** | **GDMCC 3.1267** | **PX206619** | **PX436188** | **PX654198** | **PX024416** | **PX257449** |
| ***C. tropicale*** | **GDMCC 3.1265** | **PX229582** | **PX436190** | **PX654196** | **PX024418** | **PX501982** |

(Continues)

Note: BRIP: The Building Respect for Intellectual Property Database Project. CBS: Centraalbureau voor Schimmelcultures. CFCC: China Forest Certification Council. CGMCC: China General Microbiological Culture Collection Center. CMM: Culture Collection of Phytopathogenic Fungi “Prof. Maria Menezes”, Universidade Federal Ruralde Pernambuco, Recife, Brazil. CPC: Cooperative Patent Classification. CS: Australian National Algae Culture Collection, ANACC Castray Esplanade, Hobart, Tasmania. GDMCC: Guangdong Microbial Culture Collection. GZAAS: Guizhou Academy of Agricultural Sciences. ICMP: International Collection of Microorganisms from Plants. IMI: International Mycological Institute. LC: LC Culture Collection (a personal culture collection of Lei Cai, housed in the Institute of Microbiology, Chinese Academy of Sciences). MAFF: Ministry of Agriculture, Forestry and Fisheries. MFLUCC: Mae Fah Luang University Culture Collection, Chiang Rai, Thailand. NBRC: ‌NITE Biological Resource Center. NN: The CB Rhizobium Collection, South Penrith Distribution Centre. VPRI: Victorian Plant Pathology Herbarium.
