# Supplementary material for: Occurrence and identification of Colletotrichum species in mangrove-associated anthracnose
Source: PeerJ. 2026 Jun 3;14:e21307. doi: 10.7717/peerj.21307 (PMC13242191; doi:10.7717/peerj.21307)
Supplement: Supplemental Information 2 — Note: The data are presented as mean ± standard deviation, and the lowercase letters of each treatment group are expressed as significant differences (p < 0.05). [file peerj-14-21307-s002.docx]

**Table S2 |** Pathogenicity of fungal on different hosts

| **Host** | **Lesion area(cm^2^)** | | | | | | | |
| --- | --- | --- | --- | --- | --- | --- | --- | --- |
|  | ***C. endophyticum*** | | ***C. karsti*** | | ***C. pandanicola*** | | ***C. proteae*** | ***C. tropicale*** |
|  | **GDMCC 3.1264** | **GDMCC 3.1271** | **GDMCC 3.1268** | **GDMCC 3.1266** | **GDMCC 3.1269** | **GDMCC 3.1270** | **GDMCC 3.1267** | **GDMCC 3.1265** |
| *Aegiceras corniculatum* | 0.52±0.04^a^ | 0.30±0.01^b^ | 0.18±0.03^ef^ | 0.26±0.03^c^ | 0.24±0.02^cd^ | 0.21±0.03^de^ | 0.15±0.02^f^ | 0.34±0.03^b^ |
| *Bruguiera gymnorrhiza* | - | 0.17±0.03^b^ | - | - | 0.58±0.05^a^ | - | - | - |
| *Excoecaria agallocha* | 1.05±0.05^a^ | 0.79±0.06^b^ | 0.31±0.05^e^ | 1.06±0.05^a^ | 0.36±0.06^de^ | 0.41±0.05^cd^ | 0.09±0.03^f^ | 0.48±0.07^c^ |
| *Rhizophora stylosa* | 16.17±0.69^a^ | 10.21±0.20^c^ | 2.44±0.09^d^ | 12.34±0.35^b^ | 0.68±0.12^f^ | 0.35±0.03^f^ | 1.79±0.13^e^ | 10.62±0.38^c^ |
| *Sonneratia apetala* | 0.88±0.03^b^ | 1.91±0.23^a^ | 0.68±0.03^c^ | 0.23±0.02^e^ | 0.45±0.04^d^ | 1.84±0.19^a^ | 0.30±0.02^de^ | 0.98±0.06^b^ |

Note: The data are presented as mean ± standard deviation, and the lowercase letters of each treatment group are expressed as significant differences(p <0.05).
